# GNR: Genetic-Embedded Nuclear Reaction Optimization with F-Score Filter for Gene Selection in Cancer Classification

**DOI:** 10.3390/ijms26157587

**Published:** 2025-08-06

**Authors:** Shahad Alkamli, Hala Alshamlan

**Affiliations:** Department of Information Technology, College of Computer and Information Sciences, King Saud University, P.O. Box 51178, Riyadh 11543, Saudi Arabia; 444203620@student.ksu.edu.sa

**Keywords:** gene selection, cancer classification, microarray data, Nuclear Reaction Optimization, uniform crossover, F-score filter, hybrid metaheuristic

## Abstract

The classification of cancer based on gene expression profiles is a central challenge in precision oncology due to the high dimensionality and low sample size inherent in microarray datasets. Effective gene selection is crucial for improving classification accuracy while minimizing computational overhead and model complexity. This study introduces Genetic-Embedded Nuclear Reaction Optimization (GNR), a novel hybrid metaheuristic that enhances the conventional Nuclear Reaction Optimization (NRO) algorithm by embedding a genetic uniform crossover mechanism into its fusion phase. The proposed algorithm leverages a two-stage process: an initial F-score filtering step to reduce dimensionality, followed by GNR-driven optimization to identify compact, informative gene subsets. Evaluations were conducted on six widely used microarray cancer datasets, with Support Vector Machines (SVM) employed as classifiers and performance assessed via Leave-One-Out Cross-Validation (LOOCV). Results show that GNR consistently outperforms the original NRO and several benchmark hybrid algorithms, achieving 100% classification accuracy with significantly smaller gene subsets across all datasets. These findings confirm the efficacy of the genetic-embedded fusion strategy in enhancing local exploitation while preserving the global search capabilities of NRO, thereby offering a robust and interpretable approach for gene selection in cancer classification.

## 1. Introduction

The early and accurate classification of cancer remains one of the most critical challenges in precision medicine [1]. With the emergence of microarray technologies, researchers have been able to monitor the expression levels of thousands of genes simultaneously, offering valuable insights into the molecular signatures of various cancers [2]. However, this abundance of information comes with a significant drawback: the curse of dimensionality. Gene expression datasets typically contain far more features (genes) than samples, making traditional machine learning models prone to overfitting and limiting their generalizability.

To address this, researchers have increasingly turned to metaheuristic optimization algorithms for gene selection [3]. These algorithms aim to identify compact subsets of informative genes that maximize classification accuracy while reducing redundancy. Among the many metaheuristic approaches proposed, Nuclear Reaction Optimization (NRO) [4] has gained attention for its ability to balance exploration and exploitation. NRO simulates the natural phenomena of nuclear fission and fusion to guide its search: the fission phase generates diverse candidate solutions through mutation and Lévy flight, while the fusion phase refines them toward optimality.

Despite its strengths, the standard NRO algorithm may encounter premature convergence, especially during the later stages of optimization, where diversity in the population can diminish. To overcome this limitation, we propose a novel extension called Genetic-Embedded Nuclear Reaction Optimization (GNR). GNR maintains the core structure of NRO but enhances its fusion phase with a Genetic Algorithm-inspired uniform crossover. With a 30% probability, the crossover operator recombines the best-known solution with a randomly selected peer, allowing each gene to be inherited from the best parent with 80% probability. This design preserves the mutation-driven exploration of fission while improving the fusion phase’s ability to refine solutions and avoid stagnation.

To evaluate the effectiveness of GNR, we apply it to six well-known microarray cancer datasets. A lightweight statistical filter is used as a preprocessing step to reduce the initial dimensionality, after which GNR is employed to identify optimal gene subsets. Classification performance is assessed using Support Vector Machines (SVMs) and Leave-One-Out Cross-Validation (LOOCV), ensuring a rigorous evaluation of the selected gene panels.

The rest of this paper is structured as follows: Section 2 outlines the background and foundations of the proposed method. Section 3 reviews related hybrid optimization approaches. Section 4 details the methodology, including preprocessing, feature filtering, and the GNR algorithm. Section 5 presents experimental results and comparisons, followed by biological relevance and conclusions.

## 2. Background

High-throughput gene expression data present significant challenges due to their high dimensionality and limited sample sizes, often leading to overfitting and high computational burden. Dimensionality reduction is commonly applied to address this, either through feature extraction [5]—which transforms the original feature space (e.g., PCA [6], LDA [7]) at the cost of interpretability—or through feature selection, which preserves the original gene features. Feature selection techniques are typically categorized into filter, wrapper, and hybrid methods. Filter methods evaluate each feature independently using statistical criteria [8], wrapper methods rely on classifier performance to assess feature subsets [9], and hybrid methods combine both for improved balance between accuracy and efficiency [10].

In this work, we adopt a hybrid selection strategy, first applying the F-score filter to reduce dimensionality, followed by a metaheuristic optimization stage. The optimization is conducted using an enhanced version of Nuclear Reaction Optimization (NRO), which we refer to as GNR. In GNR, we introduce an embedded genetic operator by incorporating uniform crossover into the algorithm’s fusion phase. This algorithm-level hybridization enhances exploitation, maintains diversity, and reduces the likelihood of convergence stagnation.

### 2.1. F-Score

The F-score is a straightforward and effective statistical filter technique, commonly used in feature selection for binary classification tasks. It evaluates how well a feature differentiates between two classes by comparing inter-class variance (differences between class means) to intra-class variance (variability within each class). Features with higher F-scores are deemed more significant because they offer better separation between the positive and negative classes [11].

Let a dataset D contain n samples and m features, where each sample belongs to one of two classes: positive (+) and negative (−). For a given feature $x_{i}$, let:

- $\mu_{i}^{+}$ be the mean of $x_{i}$ in the positive class,
- $\mu_{i}^{-}$ be the mean of $x_{i}$ in the negative class,
- $\mu_{i}$ be the global mean of $x_{i}$ across all samples,
- $n^{+}$ and $n^{-}$ be the number of samples in the positive and negative classes, respectively.

The F-score of feature $x_{i}$ is computed as:

(1) $$F_{i}=\frac{{\left(\mu_{i}^{+}-\mu_{i}\right)}^{2}+{\left(\mu_{i}^{-}-\mu_{i}\right)}^{2}}{\frac{1}{n^{+}-1}\sum_{k=1}^{n^{+}}{\left(x_{k,i}^{+}-\mu_{i}^{+}\right)}^{2}+\frac{1}{n^{-}-1}\sum_{k=1}^{n^{-}}{\left(x_{k,i}^{-}-\mu_{i}^{-}\right)}^{2}}$$

Here, the numerator measures between-class variance and the denominator represents within-class variance. A higher F-score indicates that the feature has a stronger ability to distinguish between the two classes.

Unlike wrapper methods that rely on classifiers, F-score is model-independent and computationally efficient, making it suitable for high-dimensional data like microarray gene expressions [12]. In this research, the F-score is used to preselect informative genes before applying the Nuclear Reaction Optimization (NRO) algorithm. This two-step process improves classification accuracy and reduces computational cost by narrowing the search space early on.

### 2.2. Nuclear Reaction Optimization (NRO)

Nuclear Reaction Optimization (NRO) is a metaheuristic optimization algorithm that draws inspiration from the physical processes of nuclear reactions, specifically fission and fusion [13]. In nature, fission involves splitting a heavy atomic nucleus into smaller fragments, while fusion refers to the merging of lighter nuclei to form a heavier one. Both reactions release a considerable amount of energy.

NRO uses these phenomena as metaphors for the optimization process. Fission encourages the exploration of the solution space by creating diverse candidate solutions, while fusion refines existing solutions to improve accuracy. This mechanism enables a dynamic balance between global exploration and local exploitation during the search for optimal results.

#### 2.2.1. Nuclear Fission Phase

In the fission phase, the algorithm generates new candidate solutions by breaking an existing one into smaller elements. This step allows the search process to cover a broader portion of the solution space, reducing the risk of becoming trapped in a local optimum. The formulation for producing a fission-based solution is as follows:

(2) $$X_{i}^{Fi}\left\{\begin{array}{c} \mathrm{Gaussian}\left(X_{best},\sigma_{1}\right)+\;\left({rand}_{n}\cdot X_{best}-P_{ne}^{s}\cdot {Ne}_{i}\right),\;\;\;\;if\;rand\;\leq \;P_{\beta },\\ \mathrm{Gaussian}\left(X_{i},\sigma_{2}\right)+\;\left({rand}_{n}\cdot X_{best}-P_{ne}^{e}\cdot {Ne}_{i}\right),\;\;\;\;\;\;\;\;\;if\;rand\;>\;P_{\beta }, \end{array}\right.$$

Here,

- $X_{i}^{Fi}:$ the novel solution produced during fission.
- $X_{best}:$ the optimal solution identified to date.
- $Gaussian\left(X,\sigma \right):$ a random variable produced around X, with σ determining the dispersion.
- $\sigma_{1},\sigma_{2}$: parameters regulating the extent of exploration (see Equations (7) and (8) below).
- ${rand}_{n}:$ a random variable introducing variability in the solution.
- $P_{ne}^{s},P_{ne}^{e}:$ mutation factors determining the scale of adjustments for subaltern and essential fission products, respectively.
- ${Ne}_{i}:$ heated neutron, calculated as ${Ne}_{i}=\frac{X_{i}}{X_{j}},$ where $X_{i}andX_{j}$ are two random solutions.
- $P_{\beta }:$ the probability governing whether subaltern or essential fission products are produced.

This method ensures a balanced search by generating some solutions near the best one $X_{best}$ for local refinement, while others are positioned farther away to promote broader exploration.

The fission process efficiency is affected by the step size, which regulates the degree of divergence of new solutions from existing ones. The step sizes are calculated with the subsequent formula:

(3) $$\sigma_{1}=\left(\frac{\log\left(g\right)}{g}\right)\cdot \left|X_{i}-X_{\mathrm{best}}\right|,$$

(4) $$\sigma_{2}=\left(\frac{\log\left(g\right)}{g}\right)\cdot \left|X_{r}-X_{\mathrm{best}}\right|.$$

Here,

- g: current generation number; the term $\frac{\log\left(g\right)}{g}$ guarantees a reduction in step sizes as iterations advance.
- $\left|X_{i}-X_{\mathrm{best}}\right|:$ the distance between current solution and best-known solution.
- $\left|X_{r}-X_{\mathrm{best}}\right|:$ the distance between random solution and best-known solution.

Initially, substantial step sizes facilitate the exploration of the solution space. Subsequently, the step sizes diminish, enabling the algorithm to narrow down the most effective solutions and achieve an optimal outcome.

Mutation factors are embedded in the fission equation to either intensify or broaden the search, helping prevent premature convergence and promoting efficient optimization. The factors are defined as follows:

(5) $$P_{\mathrm{ne}}^{s}=\mathrm{round}\left(rand+1\right)$$

(6) $$P_{\mathrm{ne}}^{e}=\mathrm{round}\left(rand+2\right)$$

Here,

- rand: a random number uniformly distributed between 0 and 1.
- The integer rounding guarantees discrete adjustment levels for the mutation process.

#### 2.2.2. Nuclear Fusion Phase

The fusion phase focuses on enhancing solutions that show promise. It consists of two components: ionization, which perturbs candidate solutions using population diversity, and fusion, which merges beneficial information from top solutions.

In the ionization step, the solution is modified by differences between randomly chosen individuals:

(7) $$X_{i,d}^{Ion}\left\{\begin{array}{c} X_{r1,d}^{Fi}\;+\;rand\;\cdot \;\left(X_{r2,d}^{Fi}-X_{i,d}^{Fi}\right),\;\;if\;rand\;\leq \;0.5,\\ X_{r1,d}^{Fi}\;-\;rand\;\cdot \;\left(X_{r2,d}^{Fi}-X_{i,d}^{Fi}\right),\;\;if\;rand\;>\;0.5, \end{array}\right.$$

Here,

- $X_{r1,d}^{Fi},{\;X}_{r2,d}^{Fi}$: components of two randomly selected fission solutions.
- $X_{i,d}^{Fi}:$ current solution.
- rand: random value for diversity.

If the difference term $X_{r2,d}^{Fi}-X_{i,d}^{Fi}$ becomes very small, the search may stagnate. To avoid this, the algorithm applies Lévy flight [14] to provide significant changes:

(8) $$X_{i,d}^{\mathrm{Ion}}=X_{i,d}^{\mathrm{Fi}}+{\left(\alpha ⊗\mathrm{Levy}\left(\beta \right)\right)}_{d}\cdot \left(X_{i,d}^{\mathrm{Fi}}-X_{\mathrm{best},d}^{\mathrm{Fi}}\right),$$

Here,

- α: a scaling factor controlling the magnitude of jumps.
- $\mathrm{Levy}\left(\beta \right)$: heavy-tailed random step size, introducing both small and large adjustments.
- ⊗: indicates element-wise multiplication.
- $X_{\mathrm{best},d}^{\mathrm{Fi}}$: best-known solution in the dth dimension.

After ionization, the fusion step combines the ionized solution with guidance from high-quality solutions. There are two options for this phase:

(9) $$X_{i}^{\mathrm{Fu}}=X_{i}^{\mathrm{Ion}}+rand\cdot \left(X_{r1}^{\mathrm{Ion}}-X_{\mathrm{best}}\right)+rand\cdot \left(X_{r2}^{\mathrm{Ion}}-X_{\mathrm{best}}\right),$$

Here,

- $X_{i}^{\mathrm{Fu}}$: refined solution after fusion.
- $X_{\mathrm{best}}:$ best-known solution guiding the search.
- $X_{r1}^{\mathrm{Ion}},X_{r2}^{\mathrm{Ion}}$: ionized solutions selected for comparison.
- rand: random value for diversity.

Or, if diversity is insufficient:

(10) $$X_{i}^{\mathrm{Fu}}=X_{i}^{\mathrm{Ion}}+\alpha ⊗\mathrm{Levy}\left(\beta \right)⊗\left(X_{i}^{\mathrm{Ion}}-X_{\mathrm{best}}^{\mathrm{Ion}}\right),$$

These fusion mechanisms help the algorithm either intensify the search around elite solutions or leap toward unexplored areas when progress slows.

In summary, the NRO algorithm leverages the interplay between nuclear fission and fusion processes to navigate the search space efficiently. By mimicking principles from nuclear physics, it adapts its search behavior to explore diverse areas and steadily move toward optimal solutions. This makes it a robust and dependable method for addressing complex optimization problems.

The explanations in Section 2.2.1 and Section 2.2.2 are adapted from our earlier study on applying NRO to gene selection [4].

## 3. Related Works

Gene selection for cancer classification has been widely addressed using metaheuristic optimization algorithms due to their ability to navigate high-dimensional search spaces effectively. Among these, a growing body of work has focused on enhancing traditional metaheuristics by embedding genetic operators, such as crossover and mutation, to improve convergence and maintain diversity. This embedded strategy allows algorithms to balance exploration and exploitation more efficiently, particularly in gene expression datasets where sample sizes are limited and feature redundancy is common. The following studies illustrate various approaches that incorporate genetic mechanisms into metaheuristic frameworks for gene selection.

Li et al. [15] integrate genetic-algorithm operators into particle swarm optimization (PSO) to create a hybrid PSO/GA for gene-subset selection. PSO supplies the positional updates, while GA’s uniform crossover (rate = 0.985) and mutation (rate = 0.05) inject diversity and curb premature convergence. Using SVM with ten-fold cross-validation on leukemia, colon, and breast-cancer microarrays, the hybrid consistently outperformed standalone PSO or GA, achieving, for example, 97.2% accuracy on leukemia with only ~19 genes. The study shows that embedding GA operators within PSO improves exploration–exploitation balance and yields compact, high-discriminative gene sets.

Chuang et al. [16] proposed a hybrid gene selection method that integrates a Combat Genetic Algorithm (CGA) into Binary Particle Swarm Optimization (BPSO) for microarray classification. The embedded CGA includes uniform crossover and a mutation operator applied with a rate of 0.05, enhancing local refinement within the global PSO framework. The method was evaluated on ten public microarray datasets using 1-NN with Leave-One-Out Cross-Validation and demonstrated strong performance—achieving the lowest classification error in 9 out of 10 datasets while significantly reducing the number of selected genes. This study confirms the benefit of embedding genetic mechanisms into swarm-based algorithms to improve convergence and selection efficiency in high-dimensional biomedical data.

Alshamlan et al. [17] proposed the Genetic Bee Colony (GBC) algorithm, a hybrid method that incorporates Genetic Algorithm (GA) operators into the Artificial Bee Colony (ABC) model for gene selection from microarray data. The approach integrates uniform crossover and mutation within the ABC structure—crossover is embedded during the onlooker bee phase, while mutation is applied in the scout bee phase. The crossover probability rate (CPR) is set to 0.8 and the mutation probability rate (MPR) is 0.01, allowing controlled genetic variation to enhance convergence and maintain diversity. GBC was evaluated on six public microarray datasets and achieved superior classification accuracy and gene subset compactness compared to standard ABC and mRMR-ABC methods. This study reinforces the effectiveness of embedding GA operators into swarm-based optimizers for high-dimensional gene selection tasks.

Motieghader et al. [18] introduced a hybrid gene selection method known as GALA, which combines a Genetic Algorithm (GA) with Learning Automata (LA) to enhance search efficiency in microarray-based cancer classification. While LA is a reinforcement-based technique rather than a metaheuristic one, it is used here to guide GA-based evolution through probabilistic feedback and adaptive updates. The genetic component employs single-point crossover and order-based mutation with rates of 0.70 and 0.30, respectively. GALA was evaluated on six public microarray datasets—including Colon, ALL_AML, SRBCT, and Tumors_9—and achieved high classification accuracy (e.g., 99.81% on Colon and 100% on ALL_AML) with compact gene subsets. Although LA is not metaheuristic, this method demonstrates how GA, when coupled with adaptive learning mechanisms, can effectively balance exploration and exploitation in high-dimensional gene selection tasks.

Collectively, these studies demonstrate the effectiveness of embedding genetic operators into metaheuristic optimization frameworks for gene selection. By integrating crossover and mutation within swarm-based or evolutionary algorithms, these approaches achieve improved classification accuracy, reduced gene subsets, and enhanced convergence behavior. Building on this foundation, the present study introduces a new GA-embedded variant of Nuclear Reaction Optimization (GNR), designed to further strengthen exploitation while preserving the exploration capabilities essential in high-dimensional biomedical search spaces.

## 4. Results and Analysis

### 4.1. Dimensionality Reduction

In the preprocessing phase, four filter methods (F-score, Information Gain (IG), ReliefF, and mRMR) were independently applied to rank genes based on their relevance to the target classes. The performance of each filter was initially assessed across multiple subset sizes (50, 100, 200, 300, 400, and 500 genes) using classification accuracy as the evaluation metric. F-score achieved the highest classification accuracy across most subset sizes, closely followed by Information Gain. ReliefF and mRMR generally produced lower classification results. To further validate these observations, the gene subsets generated by each filter were also optimized using the NRO algorithm and evaluated for final classification performance. The results confirmed that IG, ReliefF, and mRMR based preprocessing led to lower accuracies after optimization compared to using F-score. Based on this evaluation, only F-score was selected for final integration with the NRO algorithm, while the other three were excluded.

### 4.2. GNR Algorithm Results

This section reports the performance of the Genetic-Embedded Nuclear Reaction Optimization (GNR) algorithm across the six microarray cancer datasets. The evaluation lists best, average, and worst accuracies, each accompanied by precision, recall, F1-score, and 95% confidence intervals to capture both peak performance and run-to-run variability. We tested fixed subset sizes from 2 to 25 genes; to keep the tables concise, we include only those sizes that altered at least one metric and we stop reporting once the model achieved perfect accuracy for the dataset in question.

Across subsets ranging from two to twenty-two genes in the Colon dataset, every additional marker incrementally improved the Support-Vector-Machine classifier. A turning point occurred at twenty-two genes: accuracy, precision, recall, and the F1-score all reached their highest observed values and remained unchanged thereafter. The result indicates that complete discrimination between tumor and normal tissue in this binary dataset requires a comparatively broad transcript panel, with each added gene delivering complementary information until class boundaries are fully resolved (Table 1).

For the acute lymphoblastic versus acute myeloid leukemia data, a three-gene subset already secured perfect accuracy, precision, recall, and F1-score; enlarging the subset to four or five genes produced identical outcomes. The rapid convergence shows that the decisive expression differences between the two leukemia subtypes are captured by a minimal signature and that further expansion simply adds redundant features (Table 2).

In the three-class Leukemia 2 dataset, performance rose step-by-step as the subset grew from two to four genes. At four genes every evaluation metric reached its ceiling, after which larger subsets offered no improvement. The monotonic gains imply that each additional gene contributed a non-overlapping class-specific signal and that four genes constitute the smallest subset able to resolve all three disease phenotypes (Table 3).

Remarkably, the Lung adenocarcinoma dataset achieved optimal values for all four metrics with only two genes and the results remained identical when extra genes were introduced. This outcome suggests that a very limited number of transcripts dominate the discriminative structure of the data, allowing the optimization process to attain flawless classification without risk of over-parameterization (Table 4).

In the Lymphoma dataset, a two-gene subset was already capable of yielding perfect accuracy in the best trial, yet the average and worst-case accuracies remained slightly lower, indicating run-to-run variability. When a third gene was added, this variance disappeared: every repeat achieved 100% accuracy, precision, recall, and F1-score, and a fourth gene offered no further improvement. Investigators therefore may select either the ultra-compact two-gene panel, accepting minor stability loss, or the three-gene panel, which guarantees reproducible, maximized performance across all evaluations (Table 5).

The four-class Small Round Blue Cell Tumor dataset exhibited the most pronounced incremental pattern. A two-gene subset yielded moderate performance; adding a third and fourth gene produced substantial gains and a five-gene subset finally delivered perfect scores across all metrics. The orderly improvements confirm that the embedded crossover in the GNR algorithm can continue to integrate informative genes until every residual misclassification is eliminated (Table 6).

### 4.3. Comparative Analysis

To assess the effectiveness of the GNR algorithm, we compare its performance with the original NRO algorithm and several existing hybrid gene selection methods. The comparison focuses on classification accuracy and the number of selected genes.

As shown in Table 7, in the comparative performance of GNR and its predecessor NRO across all six microarray benchmarks, the Genetic-Embedded Nuclear Reaction Optimization (GNR) achieved 100% classification accuracy with gene panels ranging from two (lung, lymphoma) to twenty-two transcripts (colon). When the same datasets were evaluated with the original NRO, perfect accuracy persisted only on the three easier tasks—Leukemia 1, Lung, and Lymphoma—where the decision surfaces are already sharply defined. In the remaining, more heterogeneous datasets the shortfall was modest but systematic: NRO lagged by 1.6 percentage points on Colon, by 1.4 points on Leukemia 2, and by 2.4 points on SRBCT.

Table 8 compares GNR with nine other two-metaheuristic hybrids (SIW-APSO, HHO-GWO, iBABC-CGO, HHO-GRASP, GBC, QMFOA, PSO-GA, GALA, and PCC-GA). Accuracy is the common metric and the number of genes each method keeps appears in parentheses, so the table shows at a glance how well every algorithm balances prediction quality with subset compactness across the six public microarray cancer datasets.

GNR is the only method that reaches 100 percent on every dataset. It does so with exceptionally small panels on five tasks (3 genes for Leukemia 1, 4 for Leukemia 2, 2 for Lung, 2 for Lymphoma, and 5 for SRBCT). No competitor delivers the same mix of flawless accuracy and parsimony. HHO-GWO and GBC also report perfect scores on five datasets but require more genes across the board and both fall short on Colon. QMFOA achieves 100 percent wherever it is evaluated, yet its panels range from 20 to 32 genes. Algorithms that produce the leanest signatures, such as iBABC-CGO (≈2–8 genes) and SIW-APSO (5–13 genes), do so at the cost of missing perfect accuracy on one or more datasets.

Taken together, the comparison positions GNR as the only algorithm that attains perfect accuracy everywhere and it already keeps the gene lists extremely short on five of the six datasets. The lone outlier is the Colon set, where GNR still needs 22 genes to stay flawless. Shrinking that count is inherently difficult: the Colon matrix contains just 62 samples but 2 000 expression features, and the tumor-versus-normal signal is spread thinly across many moderately informative genes instead of being dominated by a few highly discriminative ones. Because each gene contributes only a fragment of the separating pattern, removing even a single marker can expose borderline cases and break the perfect decision boundary. Achieving the same accuracy with fewer genes will therefore require an exceptionally precise pruning step that can eliminate only those genes whose information is fully redundant while retaining the collective signal needed to distinguish the classes.

### 4.4. Biological Relevance

Table 9 presents the predictive genes identified by the GNR algorithm that achieved 100% classification accuracy across the six microarray datasets. The biological relevance of these genes was not explored in depth in this study. However, a preliminary check using the “GeneCards” database [26] indicated that many of the selected genes are associated with cancer-related functions or processes. Because this research focuses primarily on the design and evaluation of a computational gene selection method, detailed functional analysis and interpretation of individual genes are considered beyond its current scope and are better suited for future work by domain experts in biomedical research.

## 5. Materials and Methods

This study presents an enhanced hybrid approach for gene selection in cancer classification that combines statistical dimensionality reduction with a modified version of the Nuclear Reaction Optimization (NRO) algorithm. The proposed method introduces two key improvements over previous work: (1) dimensionality reduction is streamlined by using only the F-score filter, based on prior findings that demonstrated its superior compatibility with NRO; and (2) the optimization stage is extended by embedding a genetic crossover operator within the nuclear fusion phase of NRO, resulting in the proposed Genetic-Embedded Nuclear Reaction Optimization (GNR) algorithm. This modification is designed to enhance exploitation and prevent stagnation during the search process.

**Figure 1:**
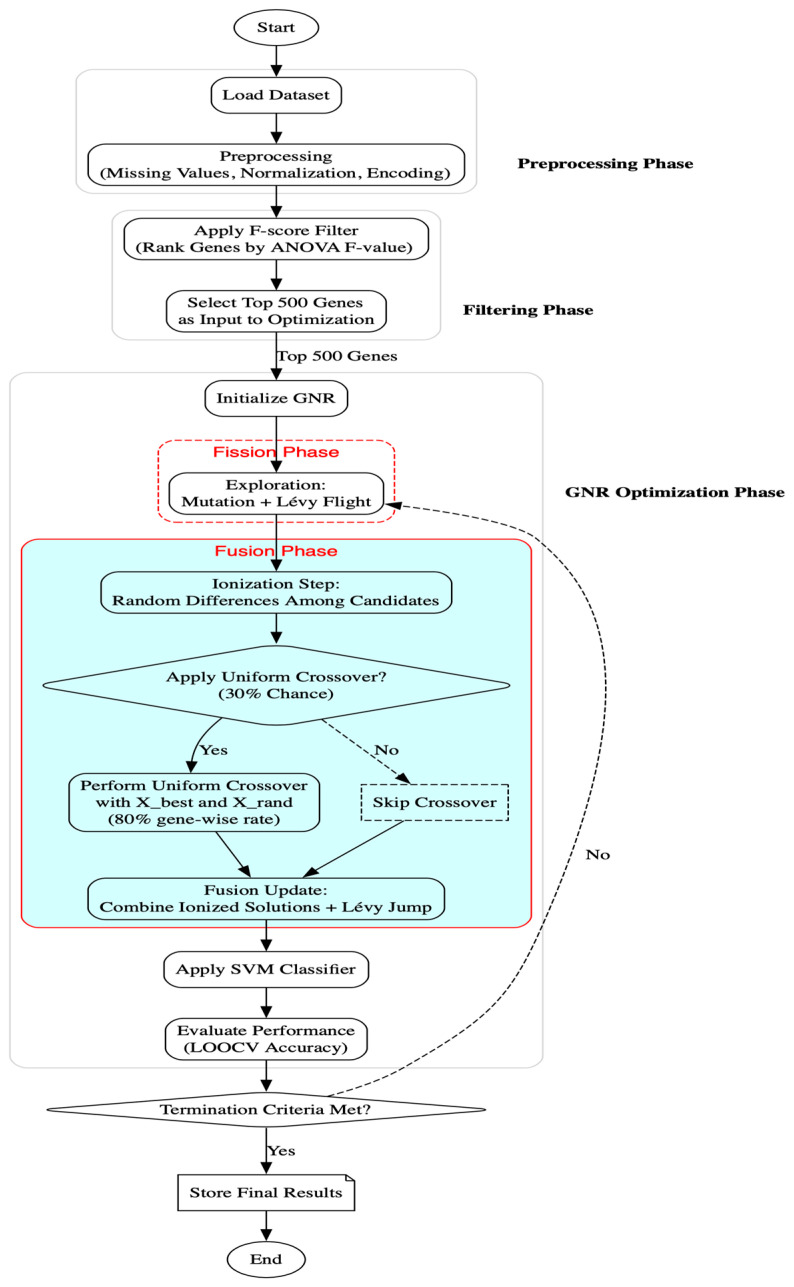
Flowchart of the methodology.

The overall methodology involves dataset preprocessing, F-score-based filtering, optimization using GNR, classification using Support Vector Machines (SVMs), and performance evaluation via cross-validation. All experiments were implemented in Python (version 3.x), using numpy and pandas for data manipulation, scipy.io.arff for dataset loading, and scikit-learn for filtering, normalization, and classification. The GNR algorithm was developed from scratch based on the mathematical foundations of NRO, including nuclear fission, fusion, Lévy flights, and mutation operators, with the new addition of a probabilistic uniform crossover mechanism embedded in the fusion phase. Figure 1 illustrates the complete workflow, from preprocessing to optimization and evaluation.

### 5.1. Dataset and Preprocessing

To evaluate the effectiveness of the proposed hybrid gene selection method, we selected six benchmark microarray cancer datasets that are widely used in bioinformatics research. These datasets include both binary and multiclass classification tasks, providing a diverse and realistic testbed for assessing model performance and generalizability. All datasets were originally published in peer-reviewed studies and are frequently used in comparative evaluations. For transparency and reproducibility, all datasets used in this study have been compiled and made publicly available in our GitHub repository: https://github.com/ShahadAlkamli/GNR.git (accessed on 1 August 2025). Version 1.0 (GitHub release tag v1.0) of the software was used for all experiments reported in this paper.

Each dataset comes with class labels that were defined in the original studies, usually based on clinical diagnosis or pathology results. For instance, the Colon dataset [27] contains samples labeled as either tumor (40 samples) or normal (22 samples). In Leukemia 1 [28], the labels distinguish between acute lymphoblastic leukemia (ALL, 47 samples) and acute myeloid leukemia (AML, 25 samples). The Lung dataset [29] separates tumor cases (86) from normal ones (10). Among the multiclass datasets, Leukemia 2 [30] includes B-cell (38), AML (25), and T-cell (9) samples. Lymphoma [31] consists of three subtypes: diffuse large B-cell lymphoma (DLBCL, 46), chronic lymphocytic leukemia (CLL, 11), and follicular lymphoma (FL, 9). In small round blue cell tumor (SRBCT) [32] , there are four tumor types, represented by numerical labels (Classes 1 through 4) with 29, 25, 18, and 11 samples.

An overview of these datasets, including the number of genes and classes, is provided in Table 10. Their variety makes them useful for evaluating how well the method performs across different types of cancer classification problems.

All datasets underwent preprocessing to ensure they were suitable for use with machine learning models. Among the datasets, only the Lymphoma dataset contained missing values, representing approximately 4.91% of its full data matrix (12,264 missing entries out of a total of 249,612). These missing values were spread across 2796 out of 4026 genes. Due to the low percentage and broad distribution of the missing data, their effect on the overall quality of the dataset was considered minimal. To maintain the structure of the data without introducing notable bias, mean imputation was employed; this involved substituting each missing value with the average expression level of the respective gene.

To address differences in gene expression scales, Z-score normalization was applied, transforming each gene’s values to have a mean of zero and a standard deviation of one. Furthermore, for multiclass classification tasks, the categorical class labels were converted to numeric form using label encoding to ensure compatibility with the classification algorithms. These preprocessing measures ensured that all datasets were fully prepared for the subsequent gene selection and classification processes.

### 5.2. F-Score-Based Dimensionality Reduction

Dimensionality reduction was performed using the F-score filter, a univariate statistical method that evaluates each gene’s ability to distinguish between classes based on ANOVA F-values. This method was selected for its computational simplicity and established effectiveness in high-dimensional data scenarios. For each dataset, F-scores were computed using the f_classif function from scikit-learn and genes were ranked accordingly. The top 500 ranked genes (k = 500) were selected as input to the optimization algorithm. This threshold was empirically chosen to ensure a strong balance between reducing dimensionality and retaining class-discriminative information.

### 5.3. Genetic-Embedded Nuclear Reaction Optimization (GNR)

To perform feature selection, we adopted the Genetic-Embedded Nuclear Reaction Optimization (GNR) algorithm, an enhanced variant of the Nuclear Reaction Optimization (NRO) algorithm. NRO is a physics-inspired metaheuristic that simulates nuclear fission and fusion processes to explore and exploit complex search spaces [13]. In our previous work [4], NRO demonstrated strong performance in selecting informative gene subsets from high-dimensional microarray data, outperforming several other metaheuristics in terms of classification accuracy and subset compactness.

However, during extended experimentation with NRO, we observed that the algorithm frequently plateaued in later generations. In many runs, the best fitness value remained unchanged across multiple consecutive generations, even though the algorithm continued iterating. This stagnation during the exploitation phase was evident from convergence curves and triggered early stopping in a significant number of trials. These findings suggested that while NRO was effective at exploration through its mutation-based fission mechanism, it lacked a strong local refinement component.

To address this limitation, we introduced a genetic operator into the fusion phase of NRO, resulting in the proposed GNR algorithm. Genetic Algorithms (GAs) are well-known for their effective exploitation strategies, particularly through crossover operations that allow the exchange of information between high-quality solutions. By embedding a uniform crossover within the fusion stage, GNR strengthens the exploitation capacity of NRO while preserving its robust exploratory behavior during fission.

In the proposed GNR algorithm, the fusion mechanism is selectively enhanced by a genetic crossover operator. After the ionization step and before applying Equation (9) or (10), a uniform crossover may be triggered. This operator is applied only if a randomly drawn value from a uniform distribution [0,1] is less than a predefined crossover probability $P_{\mathrm{CR}}=0.3$. If this condition is met, the fusion step is replaced with a genetic recombination between the current best solution $X_{best}$ and a randomly selected solution $X_{rand}$ from the population. The offspring solution is computed as:

(11) $${X_{i}}^{Fu}\left[d\right]=\left\{\begin{array}{c} X_{best}\left[d\right],\;\;\;\;\;\mathrm{if}\;rand<0.8\\ X_{rand}\left[d\right],\mathrm{otherwise} \end{array}\;for\;all\;d\in 1,2,\ldots ,D\right.$$

If the crossover condition is not satisfied, the algorithm proceeds with the original fusion mechanism using Equations (9) or (10), depending on the stagnation status. This probabilistic integration of genetic crossover improves the algorithm’s exploitation capability, promotes useful gene mixing, and reduces the likelihood of premature convergence without interfering with the fission-driven exploration process.

In the GNR algorithm, each candidate solution is encoded as a continuous vector in ${\left[0\;,1\right]}^{D}$ where *D* is the number of features (genes) after F-score filtering. The population is initialized with 500 such vectors. Optimization proceeds over a maximum of 30 generations, with early stopping enabled if no improvement is observed in the best solution for five consecutive generations. The full set of parameters used for GNR is summarized in Table 11.

To determine the optimal number of genes, we tested subsets ranging from 2 to 25 genes. For each dataset, we report the smallest number of genes that achieved 100% accuracy. This strategy highlights the minimal subset required to reach perfect classification, ensuring both performance and compactness in the selected gene panels.

This embedded genetic mechanism allows GNR to maintain NRO’s balance between exploration and exploitation while enhancing its ability to fine-tune gene subsets in later generations. The overall structure of the GNR algorithm, including initialization, dimensionality reduction, fission, fusion with crossover, fitness evaluation, and stopping conditions, is summarized in Algorithm 1.
**Algorithm 1** Genetic-Embedded Nuclear Reaction Optimization (GNR) Algorithm**Require:** Dataset D, Population size N = 500, Max generations T = 30, Early stopping patience P = 5 **Ensure:** Optimized subset of gene                       ▷ **Preprocessing and Filtering** 1:  Handle missing values (mean imputation) 2:  Normalize features using Z-score 3:  Encode categorical labels (if any) 4:  Compute F-score for each gene 5:  Select top 500 genes based on F-score                              ▷ **Initialization** 6:   **for** each subset size k ∈ {5, 10, 15} **do** 7:      Initialize population of continuous vectors xi ∈ [0, 1]D 8:      Randomly initialize each xi in population 9:      Evaluate fitness using SVM with LOOCV 10:    Set $X_{\mathrm{best}}$ ← best solution, *no_improve* ← 0                 ▷ **Fission Phase: Exploration via perturbation** 11:    **for** g = 1 to T **do** 12:    **for** each solution $x_{i}$ **do** 13:       Generate new solution ${x_{i}}^{Fi}\;$ using Equation (2) 14:       Compute dynamic step sizes using Equations (3) and (4) 15:       Apply mutation factors using Equations (5) and (6) 16:    **end for**            ▷ **Fusion Phase: Exploitation with embedded crossover** 17:    **for** each solution $x_{i}$
**do** 18:       Apply ionization via Equation (7) 19:       **if** solutions are similar **then** 20:       Apply Lévy flight adjustment using Equation (8) 21:       **end if** 22:       Generate ${x_{i}}^{Fu}$ as follows: 23:       **if** rand < 0.3 **then** 24:       Select random solution $x_{rand}$ 25:       **for** each gene d ∈ {1, . . . , D} **do** 26:          ${x_{i}}^{Fu}\;[d]$ ← if rand < 0.8: $X_{\mathrm{best}}\;[d]$; else: $x_{rand}\;[d]$ 27:       **end for** 28:       **else** 29:       Apply fusion using Equation (9) or (10) depending on similarity 30:       **end if** 31:    **end for**                ▷ **Fitness Evaluation and Best Solution Update** 32:    **for** each solution $x_{i}$
**do** 33:       Evaluate fitness via SVM + LOOCV 34:       **if**
$Fitness(x_{i})$ > $Fitness(X_{\mathrm{best}})$
**then** 35:       Update $X_{\mathrm{best}}$ ← $x_{i}$, reset *no_improve* ← 0 36:       **else** 37:       Increment *no_improve* ← *no_improve* + 1 38:       **end if** 39:    **end for** 40:    **if** *no_improve* ≥ P **then** 41:       **break**
42:    **end if** 43:    **end for** 44:   **end for**                               ▷ **Final Output** 45:   **return** best gene subset $X_{\mathrm{best}}$

### 5.4. Classification and Fitness Evaluation

Support Vector Machines (SVMs) were adopted as the classification model to evaluate the effectiveness of the gene subsets selected by the optimization algorithm. SVMs are widely recognized for their strong performance on high-dimensional gene expression data, as shown in both our previous work and in numerous studies across the bioinformatics literature. Their ability to define optimal hyperplanes in sparse feature spaces makes them particularly suitable for microarray-based cancer classification tasks.

In this study, SVMs were configured with a linear kernel and a regularization parameter of C = 1. This configuration was chosen to maintain a good balance between maximizing the decision margin and maintaining sensitivity to misclassifications. The same settings were applied across all datasets to ensure consistent model evaluation.

The fitness of the selected gene subsets was measured based on classification accuracy using Leave-One-Out Cross-Validation (LOOCV). LOOCV is considered a robust evaluation method, especially when dealing with datasets that have a small number of samples. It works by treating each sample in turn as a test instance while the remaining samples are used for training. This process continues until every sample has been used once as a test case, providing a thorough and unbiased estimate of prediction accuracy.

To compare performance, 5-fold and 10-fold cross-validation techniques were also applied to each dataset. These methods produced classification accuracies that were consistently 1 to 2 percent lower than those obtained using LOOCV. Although LOOCV was more computationally demanding, often requiring between two and ten times more time depending on the dataset, it delivered more reliable results. Since each sample in small datasets has a significant influence on learning, LOOCV was selected to minimize bias and enhance the model’s stability. Previous research in bioinformatics has also confirmed that LOOCV improves generalization and lowers the risk of overfitting in high-dimensional gene expression classification problems [20,21,22,23,33].

## 6. Conclusions

This study presented Genetic-Embedded Nuclear Reaction Optimization (GNR), a hybrid gene selection algorithm designed to improve cancer classification accuracy by enhancing the exploitation capabilities of Nuclear Reaction Optimization (NRO) through the integration of a genetic uniform crossover mechanism. By coupling this metaheuristic with a statistical F-score filter, GNR achieves a balanced trade-off between exploration and refinement in high-dimensional gene expression datasets. Experimental validation on six benchmark microarray datasets demonstrated that GNR consistently achieved perfect classification accuracy using compact gene subsets, outperforming both the original NRO and a range of competitive hybrid algorithms in terms of both accuracy and parsimony. The inclusion of the genetic operator significantly mitigated stagnation during the later optimization stages, a common limitation observed in traditional NRO implementations. These results underscore the value of hybridizing bio-inspired optimization techniques with genetic components, especially in bioinformatics tasks characterized by high feature dimensionality and limited samples.

Future work may explore further enhancements to the GNR framework by integrating biological pathway information, expanding its application to other omics data types, and conducting comprehensive biological validation of the selected gene panels. Although this study focused on microarray datasets, the GNR algorithm is compatible with RNA-seq data when appropriately normalized. Researchers interested in applying GNR to RNA-seq or other modern high-throughput data types are encouraged to adapt the framework accordingly and assess its performance in those settings.

## Figures and Tables

**Table 1:** The performance of the proposed GNR algorithm for the Colon dataset.

Dataset	Total Genes	Filtered Genes	Selected Genes	Accuracy			Precision	Recall	F1-Score	CI (95%)
				Best	Average	Worst				
**Colon**	2000	500	2	93.55%	89.46%	83.87%	93.87%	93.55%	93.61%	[88.82%, 90.11%]
			3	93.55%	90.86%	85.48%	93.87%	93.55%	93.61%	[90.40%, 91.32%]
			4	95.16%	92.15%	87.10%	95.26%	95.16%	95.18%	[91.71%, 92.59%]
			5	93.55%	92.10%	87.10%	93.62%	93.55%	93.55%	[91.73%, 92.46%]
			8	96.77%	94.09%	90.32%	97.04%	96.77%	96.80%	[93.69%, 94.48%]
			13	98.39%	95.00%	90.32%	98.43%	98.39%	98.38%	[94.57%, 95.43%]
			22	100%	95.54%	91.94%	100%	100%	100%	[95.02%, 96.05%]

**Table 2:** The performance of the proposed GNR algorithm for the Leukemia 1 dataset.

Dataset	Total Genes	Filtered Genes	Selected Genes	Accuracy			Precision	Recall	F1-Score	CI (95%)
				Best	Average	Worst				
Leukemia 1	7129	500	2	98.61%	98.47%	93.06%	98.66%	98.61%	98.62%	[98.31%, 98.63%]
			3	100%	99.26%	95.83%	100%	100%	100%	[99.00%, 99.52%]

**Table 3:** The performance of the proposed GNR algorithm for the Leukemia 2 dataset.

Dataset	Total Genes	Filtered Genes	Selected Genes	Accuracy			Precision	Recall	F1-Score	CI (95%)
				Best	Average	Worst				
Leukemia 2	7129	500	2	95.83%	93.24%	84.72%	95.93%	95.83%	95.84%	[92.67%, 93.81%]
			3	98.61%	95.74%	88.89%	98.75%	98.61%	98.64%	[95.07%, 96.41%]
			4	100%	96.85%	91.67%	100%	100%	100%	[96.40%, 97.30%]

**Table 4:** The performance of the proposed GNR algorithm for the Lung dataset.

Dataset	Total Genes	Filtered Genes	Selected Genes	Accuracy			Precision	Recall	F1-Score	CI (95%)
				Best	Average	Worst				
Lung	7129	500	2	100%	100%	100%	100%	100%	100%	100% (constant)
			3	100%	100%	100%	100%	100%	100%	100% (constant)

**Table 5:** The performance of the proposed GNR algorithm for the Lymphoma dataset.

Dataset	Total Genes	Filtered Genes	Selected Genes	Accuracy			Precision	Recall	F1-Score	CI (95%)
				Best	Average	Worst				
Lymphoma	4026	500	2	100%	98.74%	95.45%	100%	100%	100%	[98.44%, 99.04%]
			3	100%	100%	96.97%	100%	100%	100%	100% (constant)

**Table 6:** The performance of the proposed GNR algorithm for the SRBCT dataset.

Dataset	Total Genes	Filtered Genes	Selected Genes	Accuracy			Precision	Recall	F1-Score	CI (95%)
				Best	Average	Worst				
SRBCT	2308	500	2	84.34%	81.45%	73.49%	85.62%	84.34%	84.44%	[80.96%, 81.93%]
			3	96.39%	91.24%	80.72%	96.72%	96.39%	96.42%	[90.16%, 92.33%]
			4	97.59%	93.78%	84.34%	97.75%	97.59%	97.51%	[92.96%, 94.59%]
			5	100%	95.14%	86.75%	100%	100%	100%	[94.44%, 95.84%]

**Table 7:** Side-by-side summary of accuracy and gene counts for GNR versus F-NRO.

Dataset	Selected Genes	GNR	NRO
Colon	22	100%	98.39%
Leukemia 1	3	100%	100%
Leukemia 2	4	100%	98.61%
Lung	2	100%	100%
Lymphoma	2	100%	100%
SRBCT	5	100%	97.59%

**Table 8:** Comparison of GNR with other two-metaheuristic hybrid algorithms.

Algorithm	Colon	Leukemia 1	Leukemia 2	Lung	Lymphoma	SRBCT
GNR	100% (22)	100% (3)	100% (4)	100% (2)	100% (2)	100% (5)
SIW-APSO [19]	96.86% (13)	100% (5)	-	98.67% (11)	93.79% (7)	100% (7)
HHOGWO [20]	99.8% (10)	100% (11)	100% (4)	100% (3)	100% (2)	100% (12)
iBABC-CGO [21]	97.16% (2.2)	100% (2.4)	-	96.04% (7.6)	100% (2)	100% (4.8)
HHO-GRASP [22]	93.88% (7)	-	-	-	-	-
GBC [17]	98.38% (10)	100% (4)	100% (8)	100% (4)	100% (4)	100% (6)
QMFOA [23]	100% (27)	100% (30)	100% (32)	100% (20)	-	100% (23)
PSO-GA [15]	91.9% (18)	97.2% (19)	-	-	-	-
GALA [24]	99.77% (10)	-	-	-	-	99.34% (6)
PCC-GA [25]	91.94% (29)	-	100% (35)	97.54% (42)	100% (39)	100% (20)

**Table 9:** The best predictive genes that give highest classification accuracy for all microarray datasets using a GNR algorithm.

Dataset	Predictive Genes	Accuracy
Colon	T68848, L08069, X74795, U31525, L05144, T90280, X87342, R01124, D14520, R54097, T47584, T47383, T58861, R88740, T51539, U31215, T84049, H20709, T72879, R80427, M29065, X65873	100%
Leukemia 1	M75715_s_at, HG1612-HT1612_at, X95735_at	100%
Leukemia 2	M37271_s_at, D87459_at, X85116_rna1_s_at, M89957_at	100%
Lung	U53446_at, U60115_at	100%
Lymphoma	GENE1602X, GENE3621X	100%
SRBCT	gene2000, gene509, gene586, gene545, gene742	100%

**Table 10:** Statistics of microarray cancer datasets.

Microarray Dataset	Classes	Samples	Total Genes
Colon [27]	2	62	2000
Leukemia 1 [28]	2	72	7129
Leukemia 2 [30]	3	72	7129
Lung [29]	2	96	7129
Lymphoma [31]	3	62	4026
SRBCT [32]	4	83	2308

**Table 11:** Summary of key parameters used in the GNR algorithm.

Parameter	Value
Population size	500
Maximum generations	30
Early stopping rounds	5 consecutive no-improvement rounds
Number of runs	30
Crossover probability	0.3
Inheritance from best	0.8
Evaluated gene subset sizes	From 2 to 25 genes

## Data Availability

The datasets and source code used in this study are publicly available at: https://github.com/ShahadAlkamli/GNR.git (accessed on 1 August 2025).
